# Supplementary material for: Molecular mechanism of active Cas7-11 in processing CRISPR RNA and interfering target RNA
Source: eLife. 2022 Oct 3;11:e81678. doi: 10.7554/eLife.81678 (PMC9629832; doi:10.7554/eLife.81678)
Supplement: Figure 1—figure supplement 1—source data 1. [file elife-81678-fig1-figsupp1-data1.zip › Figure 1 -figure supplement 1- source data 1/Figure 2 -figure supplement 2- source data 1 .pptx]

## Slide 1
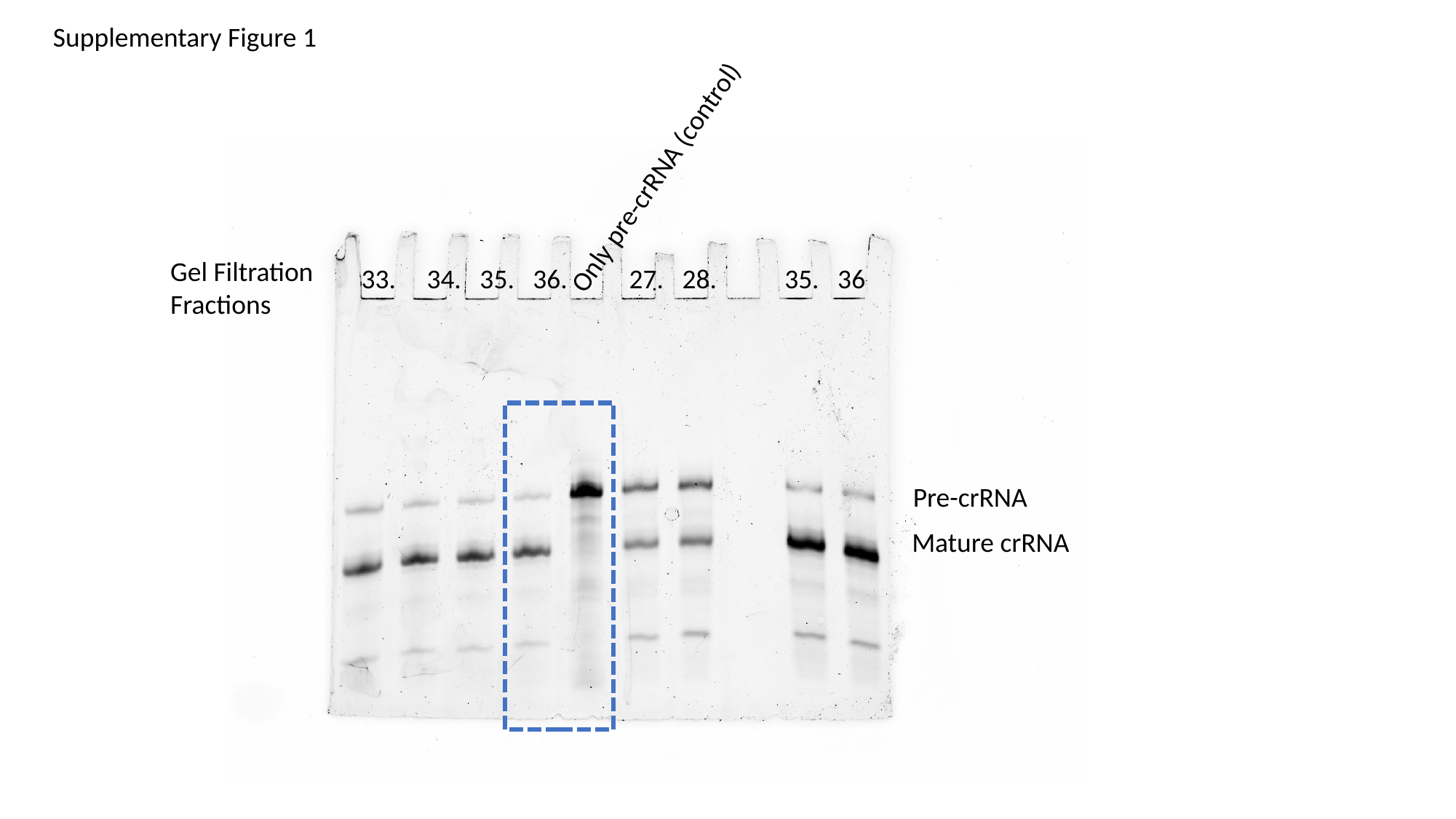

Supplementary Figure 1
Only pre-crRNA (control)
Gel Filtration
Fractions
33. 34. 35. 36. 27. 28. 35. 36
Pre-crRNA
Mature crRNA

## Slide 2
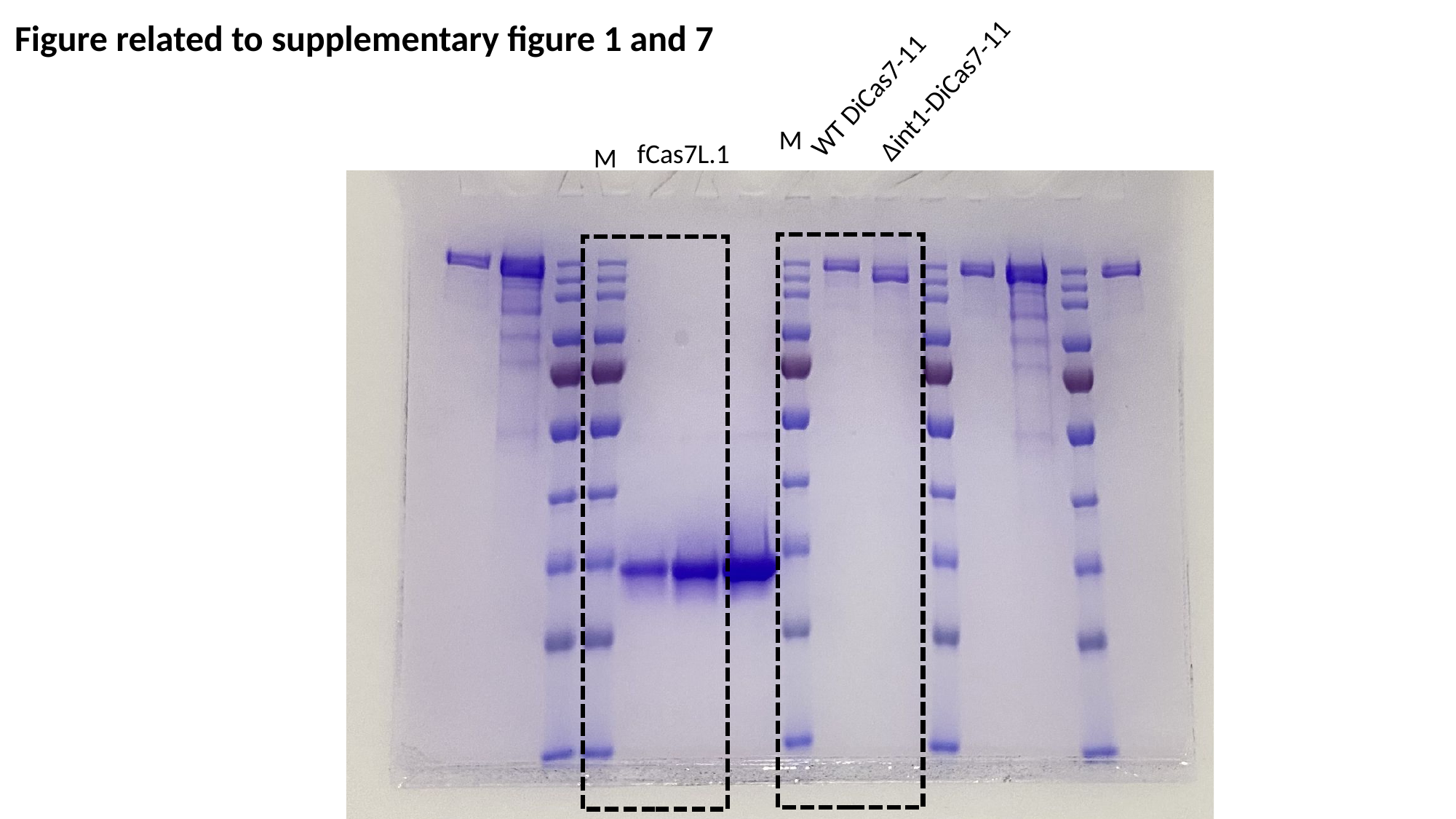

Figure related to supplementary figure 1 and 7
Δint1-DiCas7-11
WT DiCas7-11
M
fCas7L.1
M
