# Supplementary figures and images for: Molecular mechanism of active Cas7-11 in processing CRISPR RNA and interfering target RNA
Source: eLife. 2022 Oct 3;11:e81678. doi: 10.7554/eLife.81678 (PMC9629832; doi:10.7554/eLife.81678)

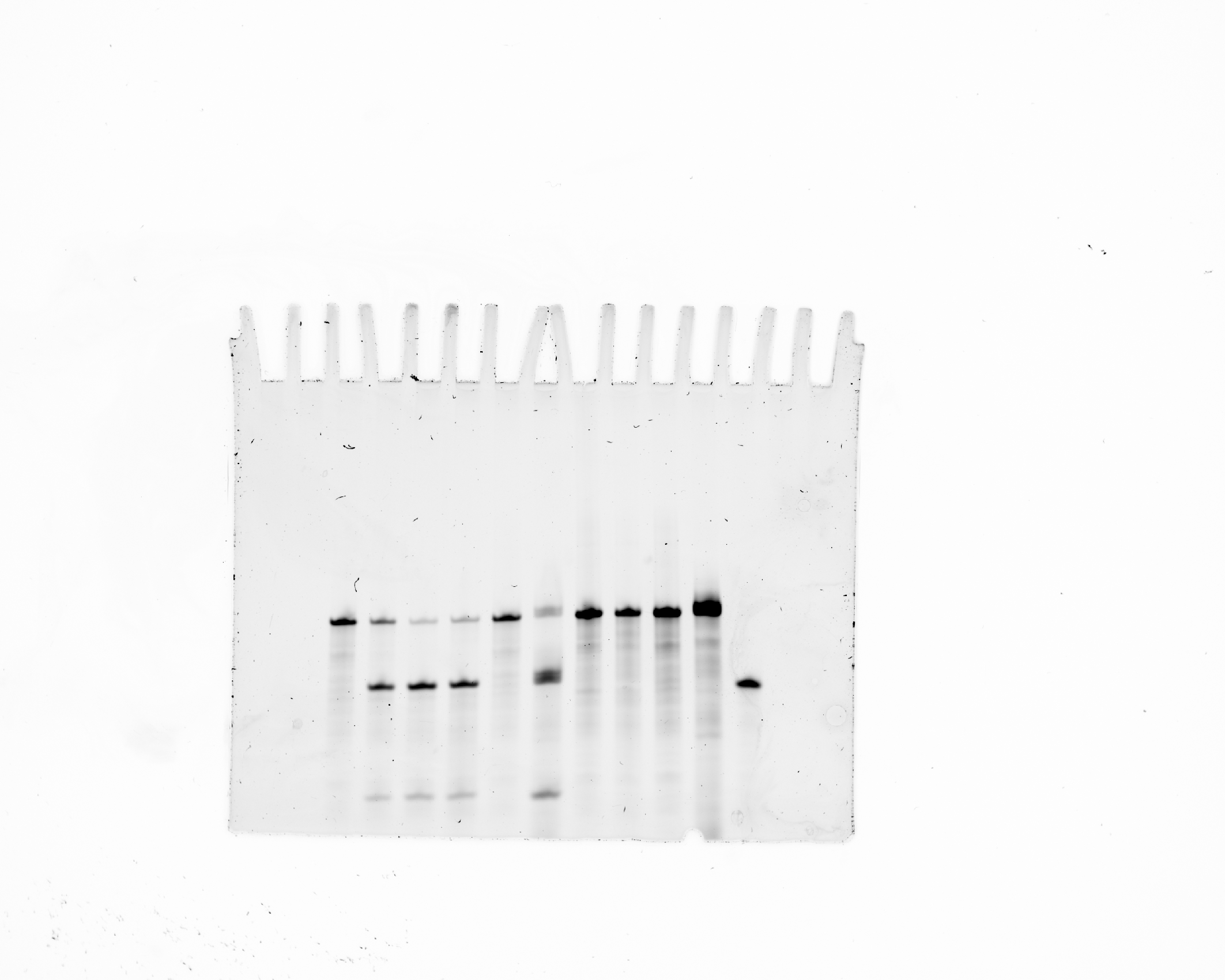

Supplement: Figure 2—source data 1. [file elife-81678-fig2-data1.zip › Figure 2 source data 1/Figure 2-source data 1 original gel.tif]

## Slide 1
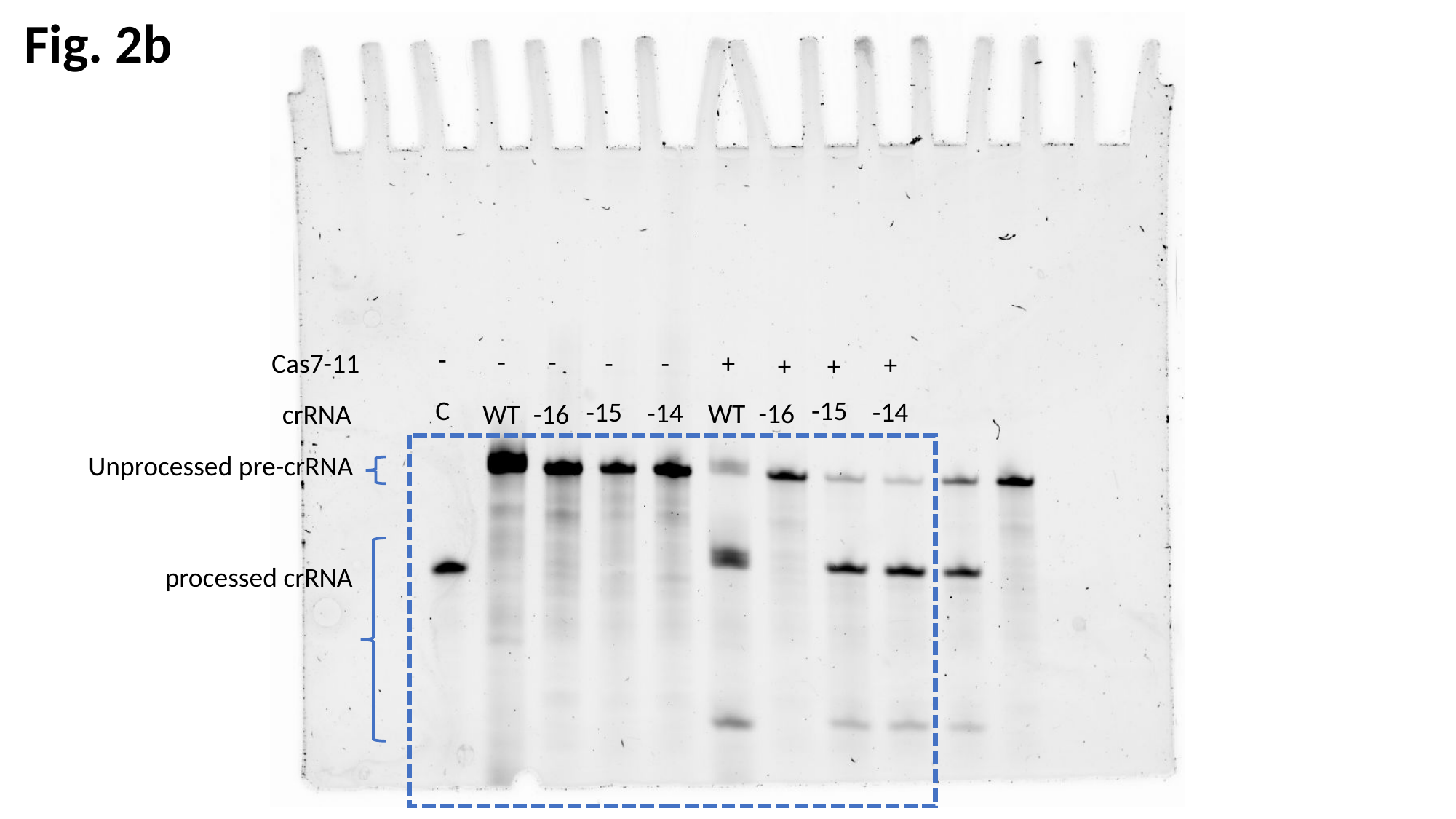

Fig. 2b
-
-
-
Cas7-11
-
-
+
+
+
+
C
-15
-15
-14
-14
WT
-16
crRNA
WT
-16
Unprocessed pre-crRNA
-
processed crRNA

Supplement: Figure 2—source data 1. [file elife-81678-fig2-data1.zip › Figure 2 source data 1/Figure 2-source data 1 .pptx]

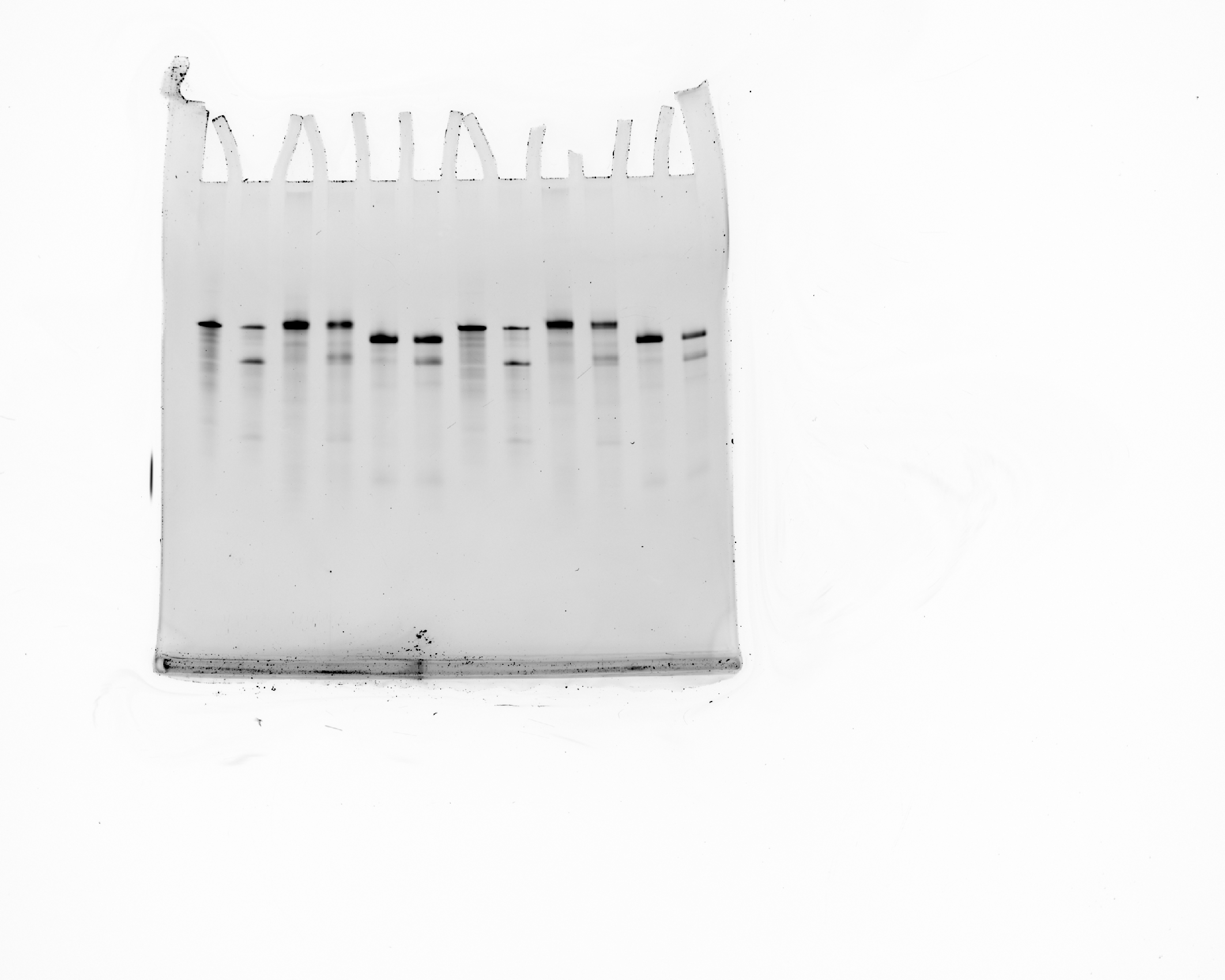

Supplement: Figure 2—source data 2. [file elife-81678-fig2-data2.zip › Figure 2 source data 2/Figure 2 -source data 2 original gel right figure.tif]

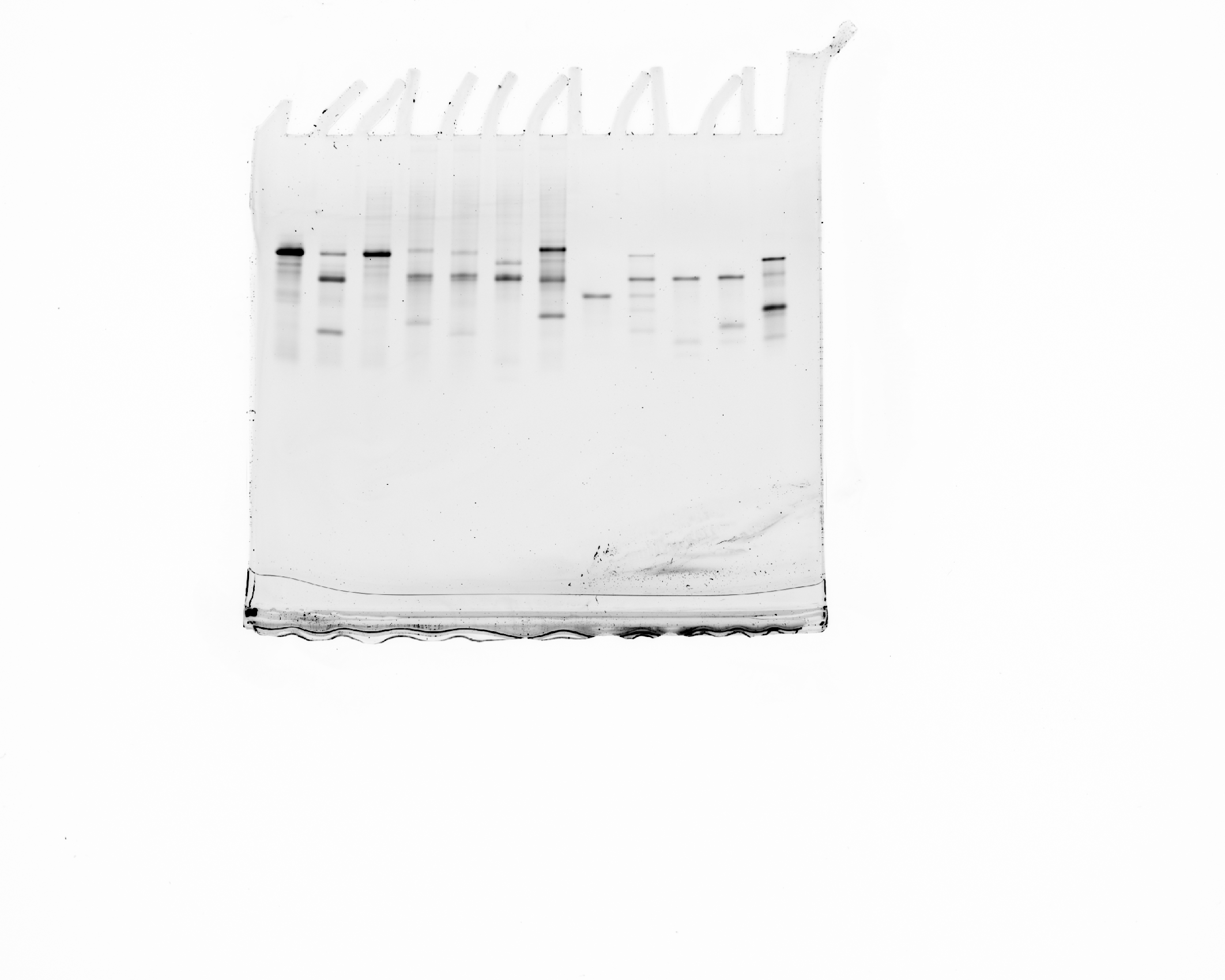

Supplement: Figure 2—source data 2. [file elife-81678-fig2-data2.zip › Figure 2 source data 2/Figure 2-source data 2 original gel left figure.tif]

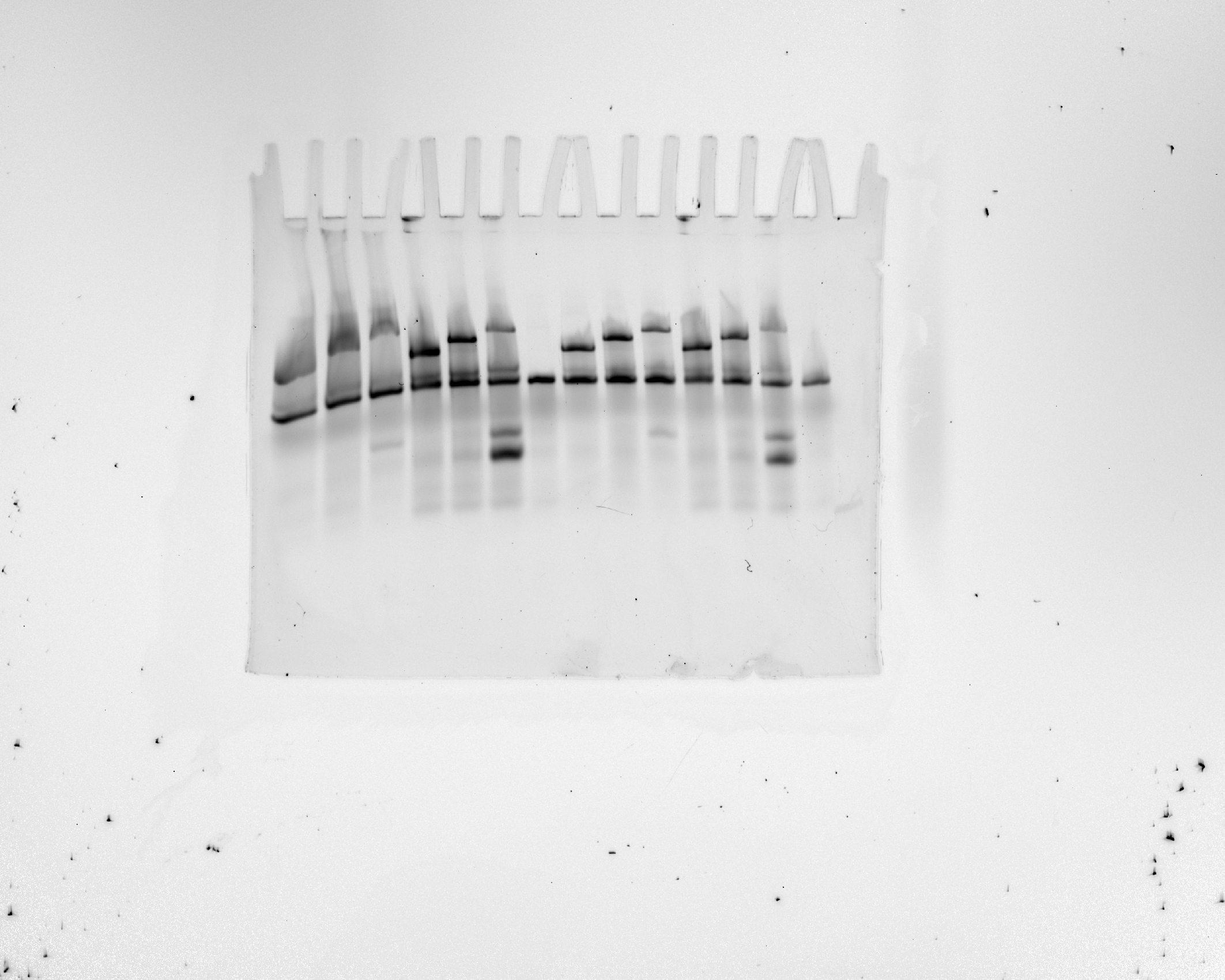

Supplement: Figure 2—source data 3. [file elife-81678-fig2-data3.zip › Figure 2 source data 3/Figure 2- source data 3 original gel.tif]

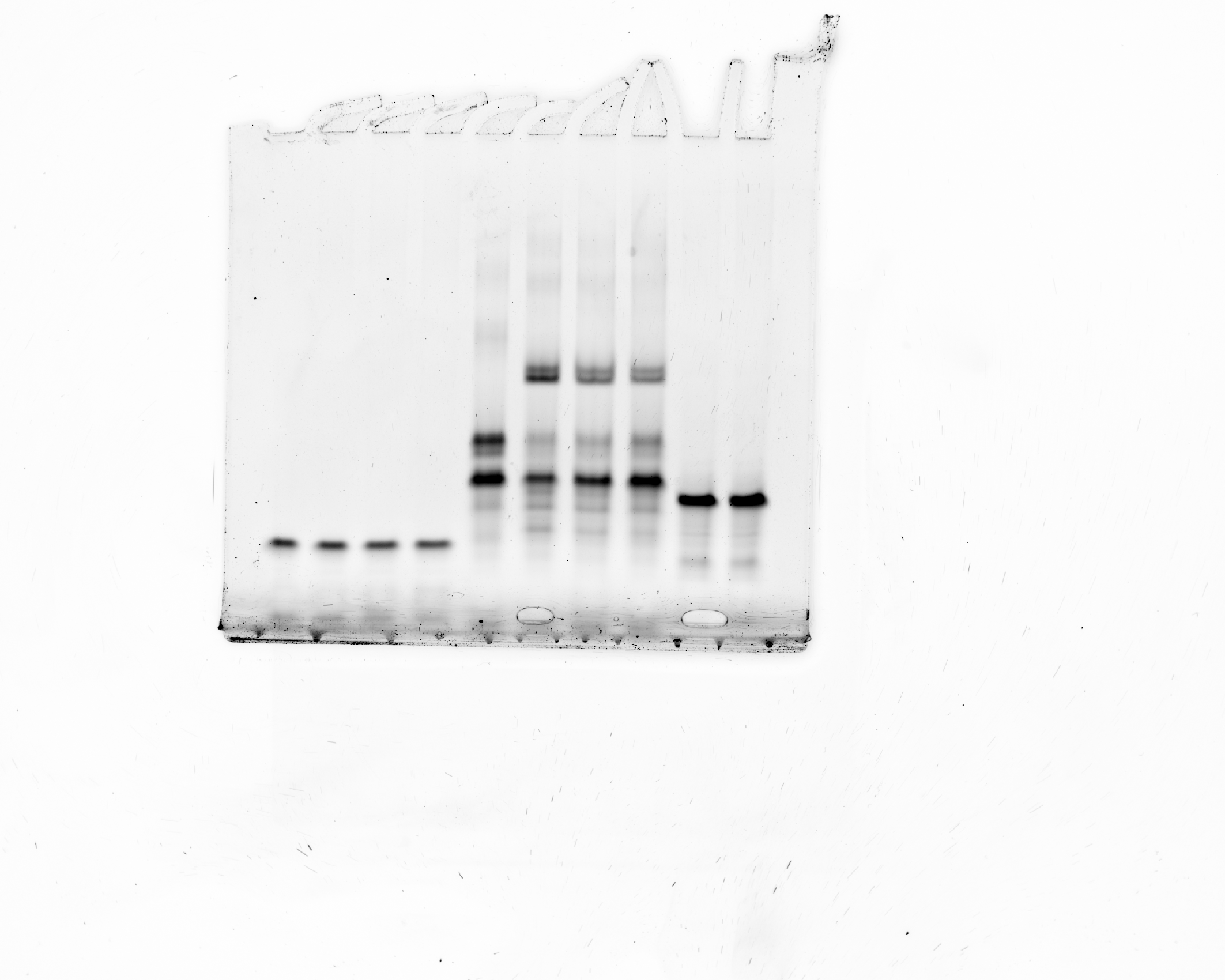

Supplement: Figure 2—figure supplement 2—source data 1. [file elife-81678-fig2-figsupp2-data1.zip › Figure 2 -figure supplement 2- source data 1 /Figure 2-figure supplement 2- source data 1 original gel.tif]

## Slide 1
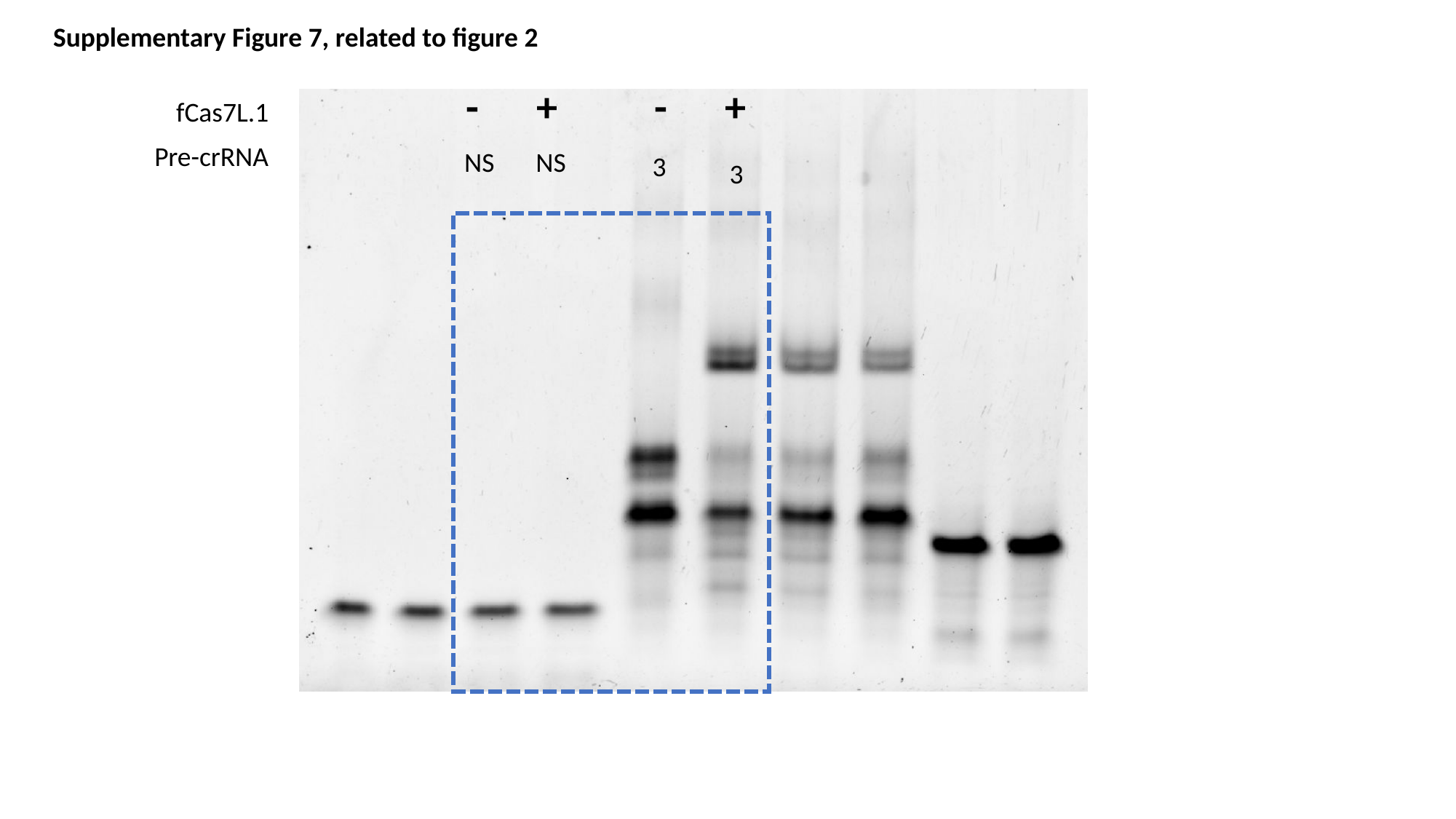

Supplementary Figure 7, related to figure 2
- + - +
fCas7L.1
Pre-crRNA
NS
NS
3
3

Supplement: Figure 2—figure supplement 2—source data 1. [file elife-81678-fig2-figsupp2-data1.zip › Figure 2 -figure supplement 2- source data 1 /Supplementary Figure 7 source data 1.pptx]

## Slide 1
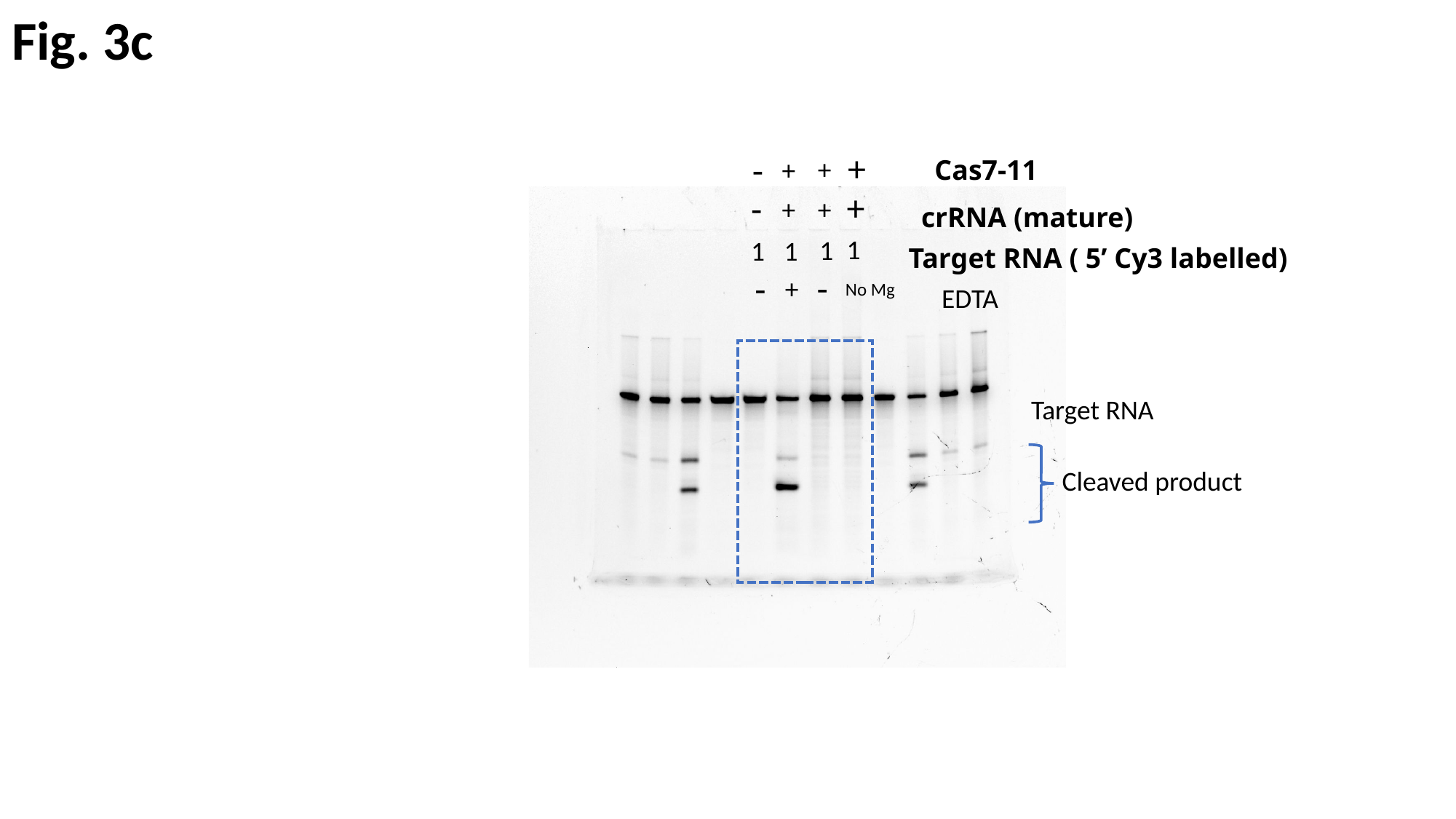

Fig. 3c
+
-
+
+
Cas7-11
+
-
+
+
crRNA (mature)
1
1
1
1
Target RNA ( 5’ Cy3 labelled)
-
-
+
No Mg
EDTA
Target RNA
Cleaved product

Supplement: Figure 3—source data 1. [file elife-81678-fig3-data1.zip › Figure 3 source data 1/Figure 3 source data 1.pptx]

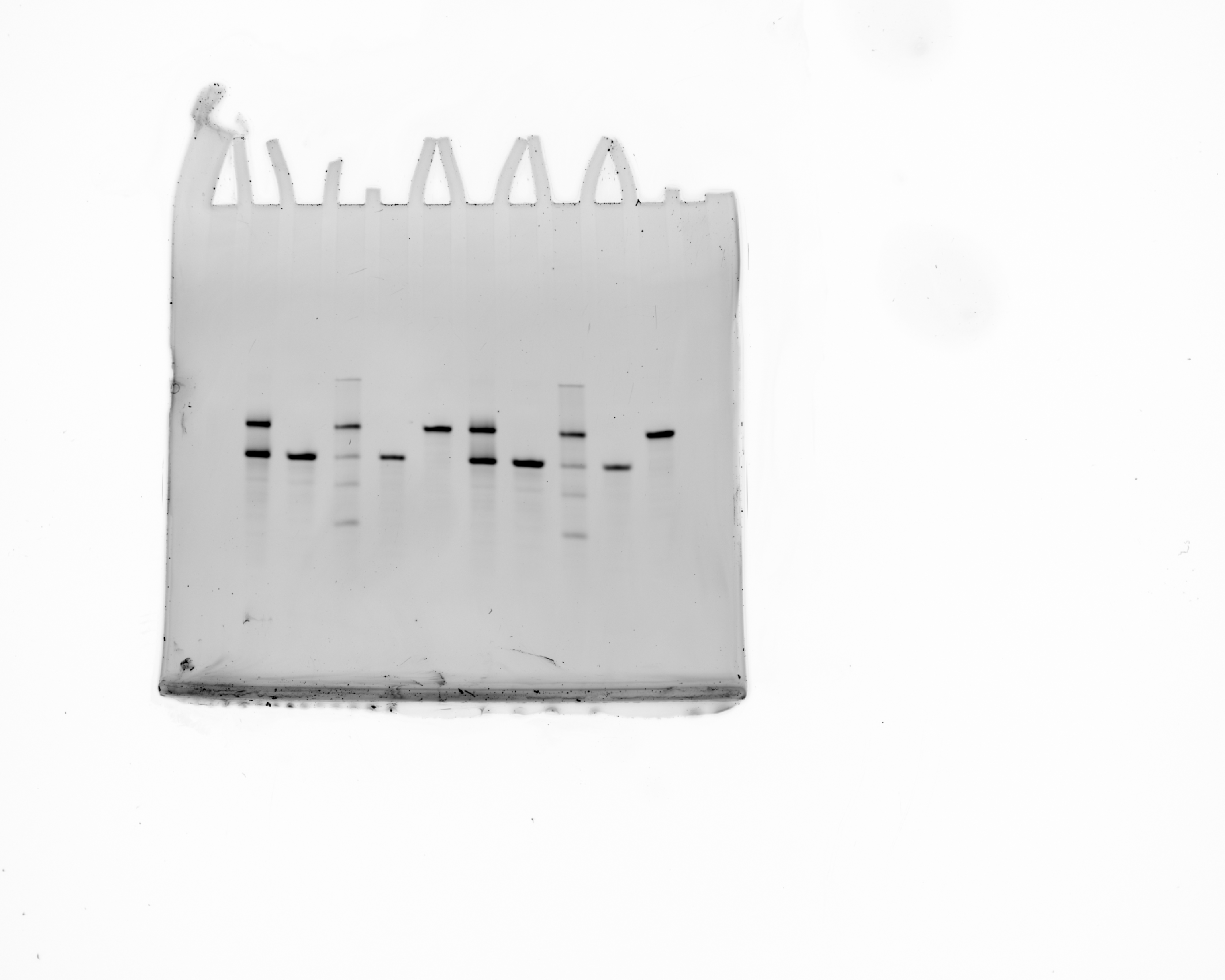

Supplement: Figure 3—source data 1. [file elife-81678-fig3-data1.zip › Figure 3 source data 1/Figure 3-source data 1 original gel.tif]

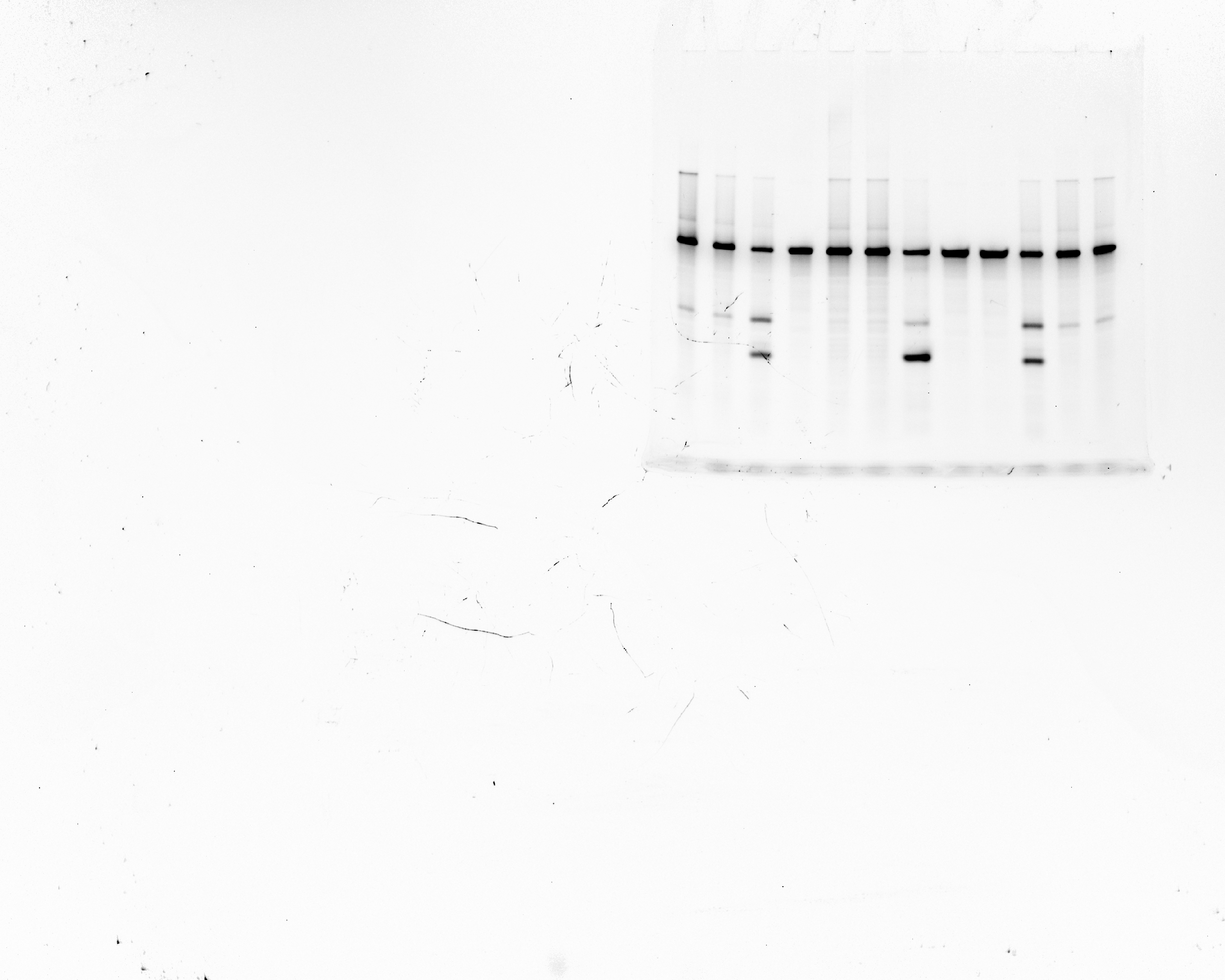

Supplement: Figure 3—source data 2. [file elife-81678-fig3-data2.zip › Figure 3 source data 2/Figure 3- source data 2 original gel.tif]

## Slide 1
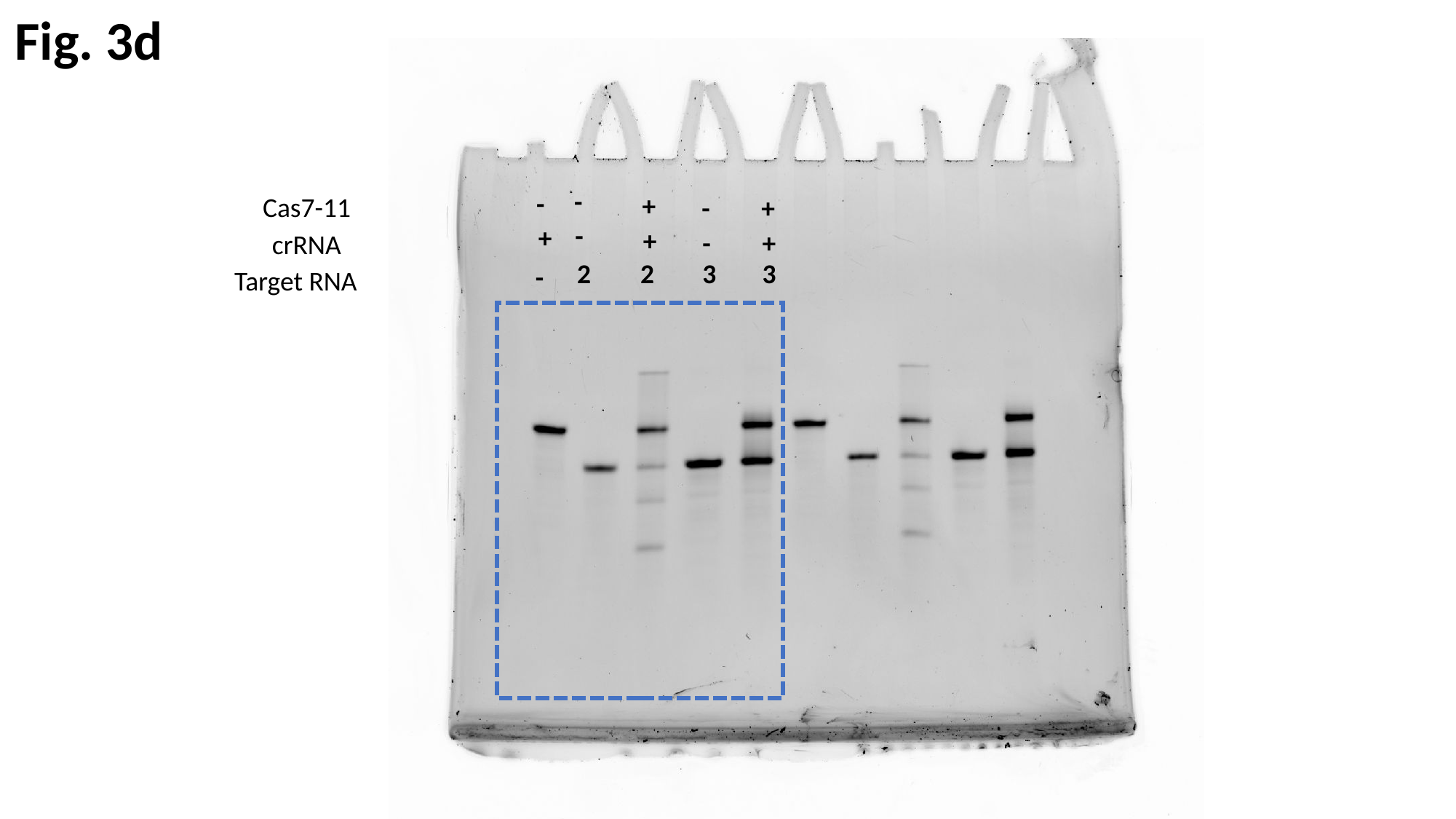

Fig. 3d
-
-
+
Cas7-11
-
+
-
+
+
-
+
crRNA
2
2
3
3
-
Target RNA

Supplement: Figure 3—source data 2. [file elife-81678-fig3-data2.zip › Figure 3 source data 2/Figure 3 source data 2.pptx]

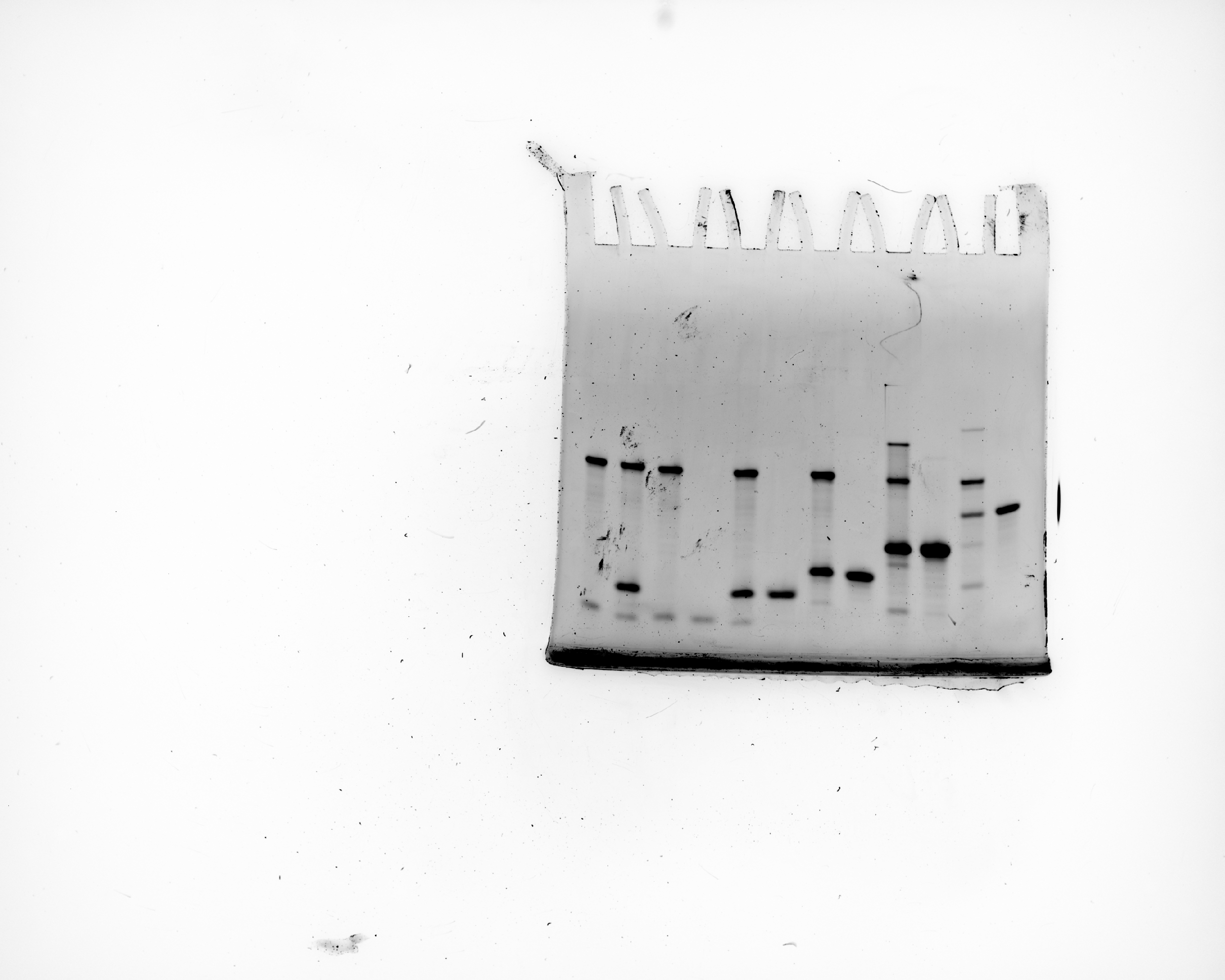

Supplement: Figure 3—source data 3. [file elife-81678-fig3-data3.zip › Figure 3 source data 3/Figure 3 source data 3 original gel.tif]

## Slide 1
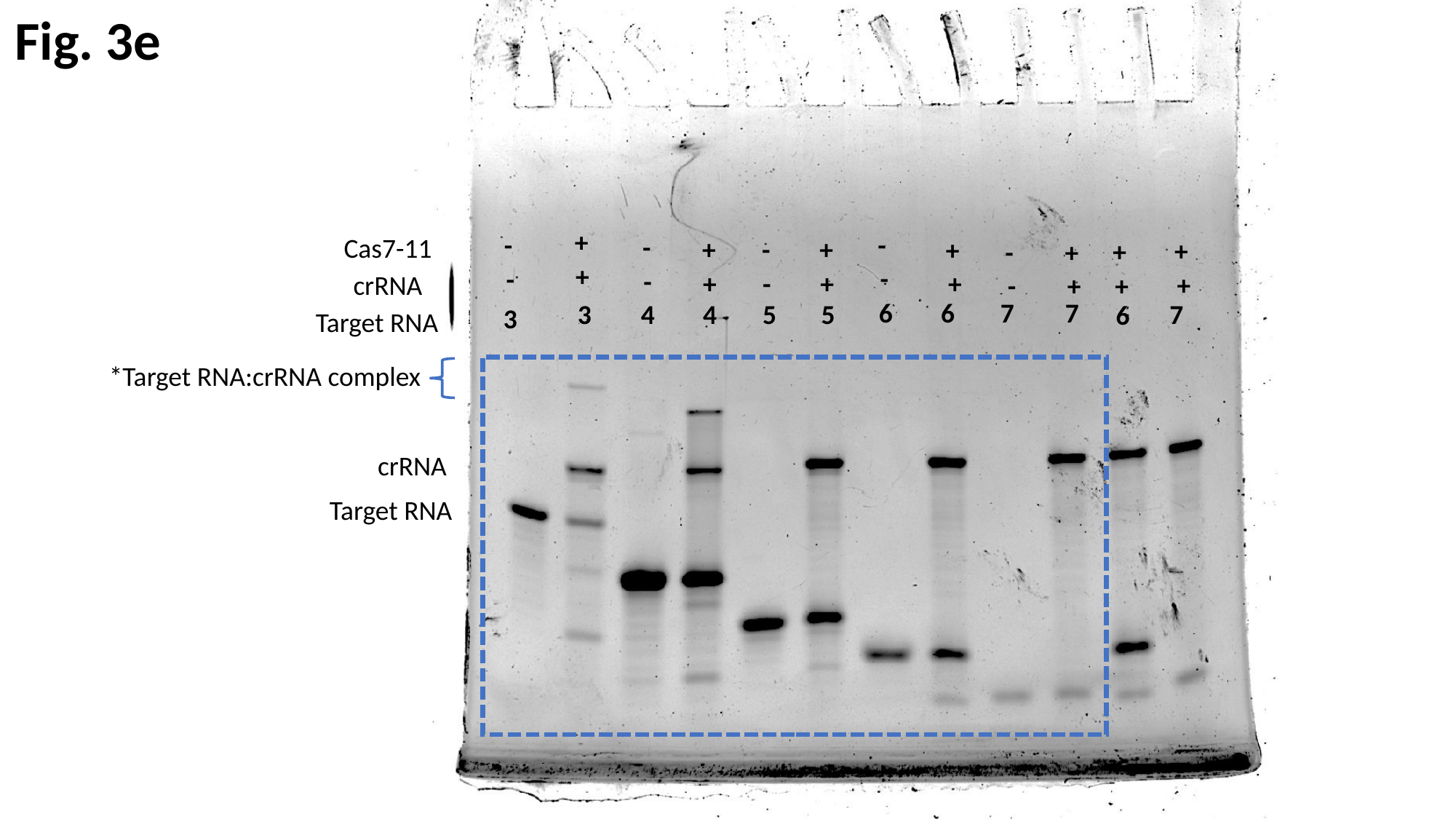

Fig. 3e
+
-
-
-
Cas7-11
+
+
-
+
+
+
-
+
+
-
-
-
+
+
-
+
+
crRNA
+
-
+
6
6
7
 7
7
3
4
4
5
5
6
3
Target RNA
*Target RNA:crRNA complex
crRNA
Target RNA

Supplement: Figure 3—source data 3. [file elife-81678-fig3-data3.zip › Figure 3 source data 3/Figure 3 source data 3.pptx]

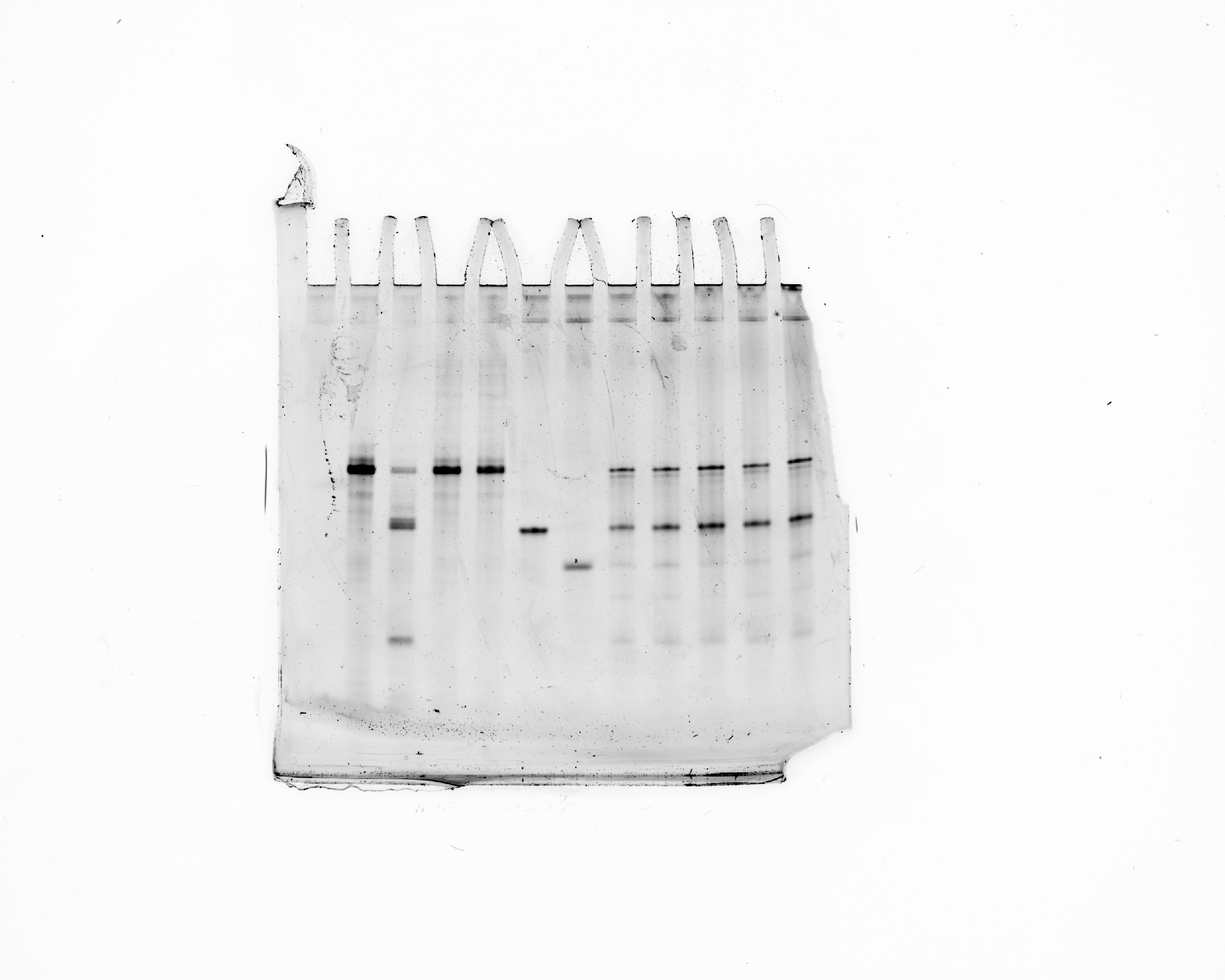

Supplement: Figure 3—source data 4. [file elife-81678-fig3-data4.zip › Figure 3 source data 4/Figure 3-source data 4 original gel.tif]
