# Supplementary material for: Molecular mechanism of active Cas7-11 in processing CRISPR RNA and interfering target RNA
Source: eLife. 2022 Oct 3;11:e81678. doi: 10.7554/eLife.81678 (PMC9629832; doi:10.7554/eLife.81678)
Supplement: Figure 2—source data 2. [file elife-81678-fig2-data2.zip › Figure 2 source data 2/Figure 2 source data 2.pptx]

## Slide 1
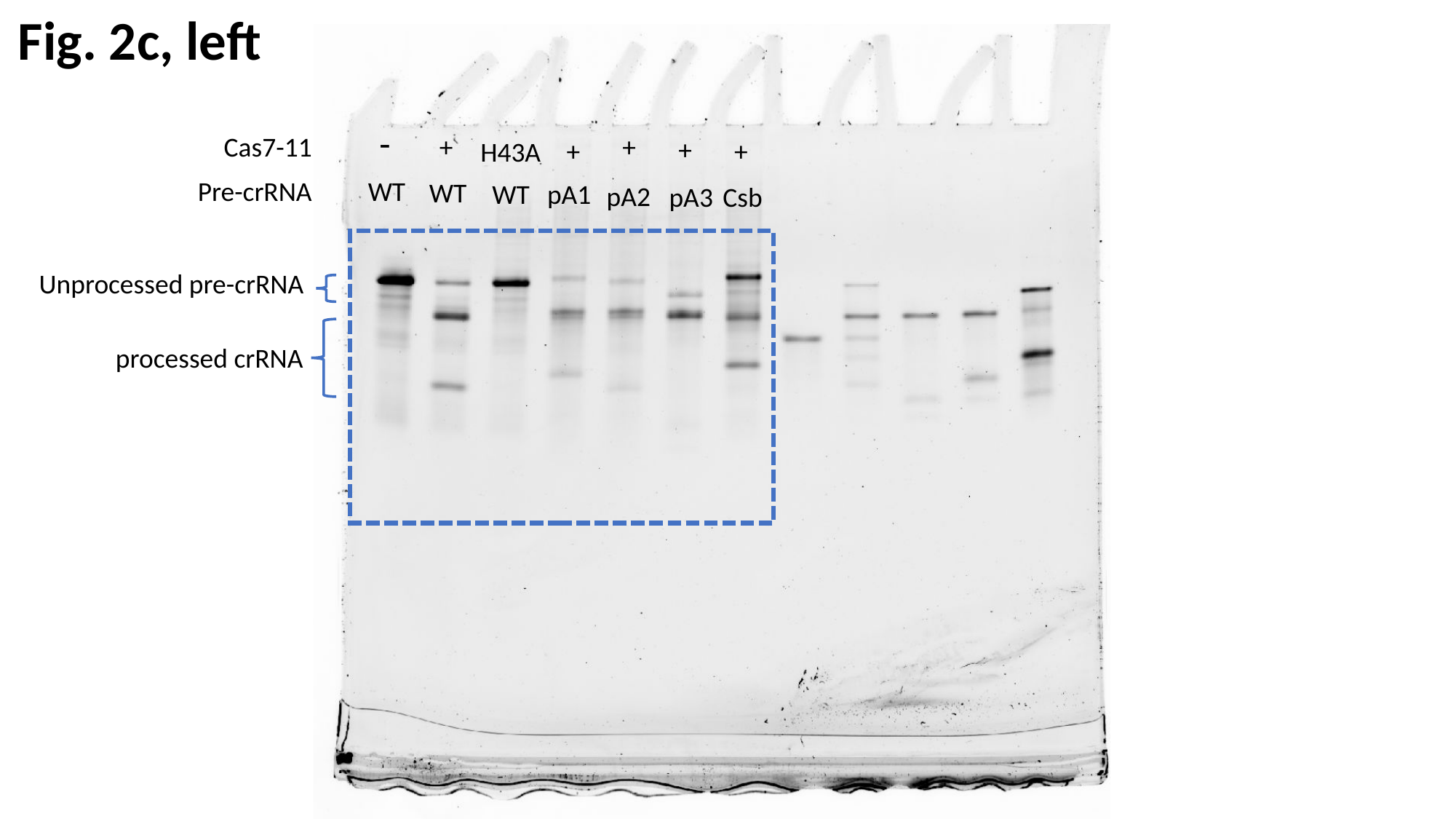

Fig. 2c, left
-
Cas7-11
+
+
+
+
+
H43A
Pre-crRNA
WT
WT
WT
pA1
pA2
pA3
Csb
Unprocessed pre-crRNA
processed crRNA

## Slide 2
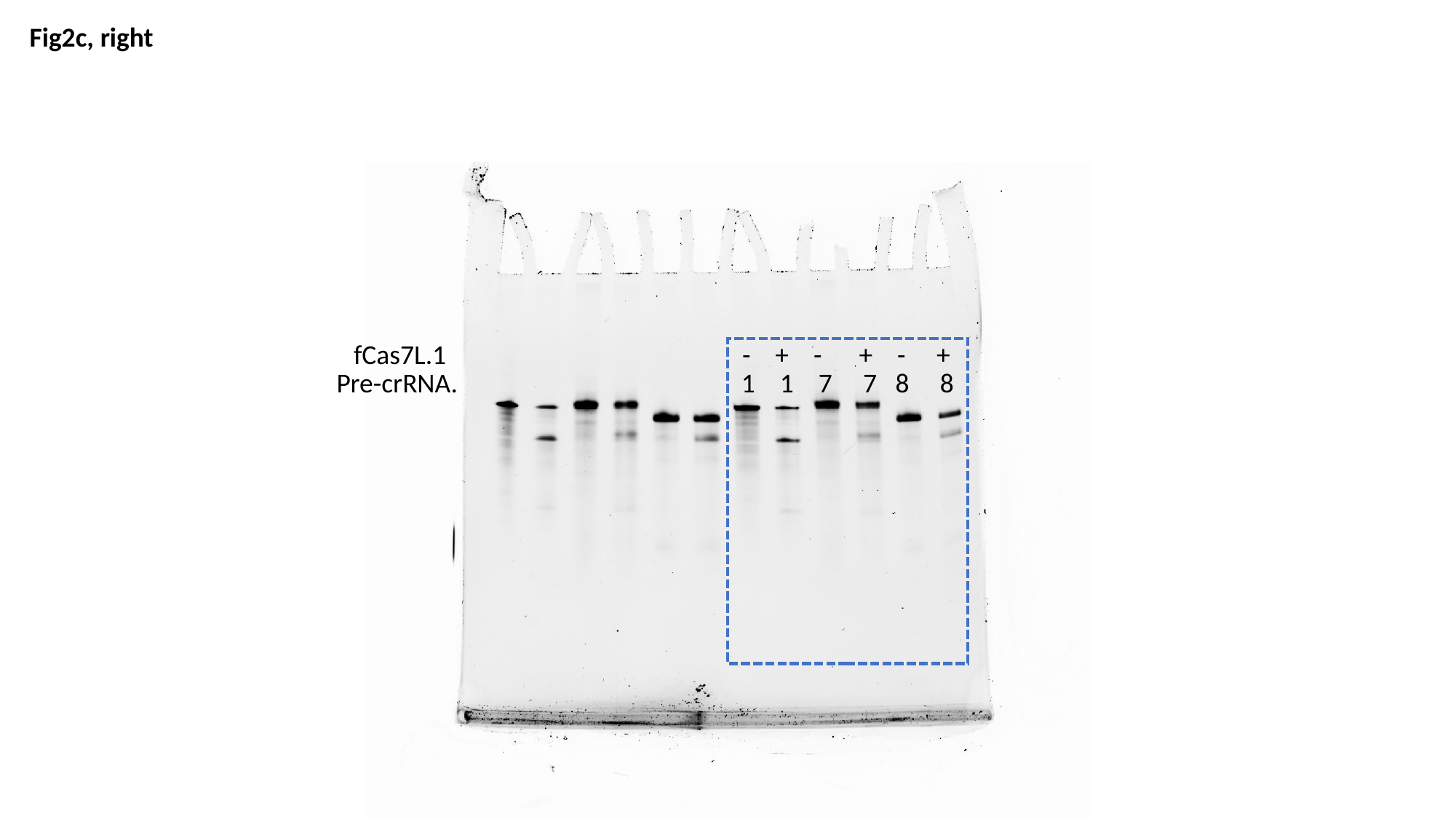

Fig2c, right
fCas7L.1 - + - + - +
Pre-crRNA. 1 1 7 7 8 8
