# Supplementary material for: Molecular mechanism of active Cas7-11 in processing CRISPR RNA and interfering target RNA
Source: eLife. 2022 Oct 3;11:e81678. doi: 10.7554/eLife.81678 (PMC9629832; doi:10.7554/eLife.81678)
Supplement: Figure 2—source data 3. [file elife-81678-fig2-data3.zip › Figure 2 source data 3/Figure 2 source data 3.pptx]

## Slide 1
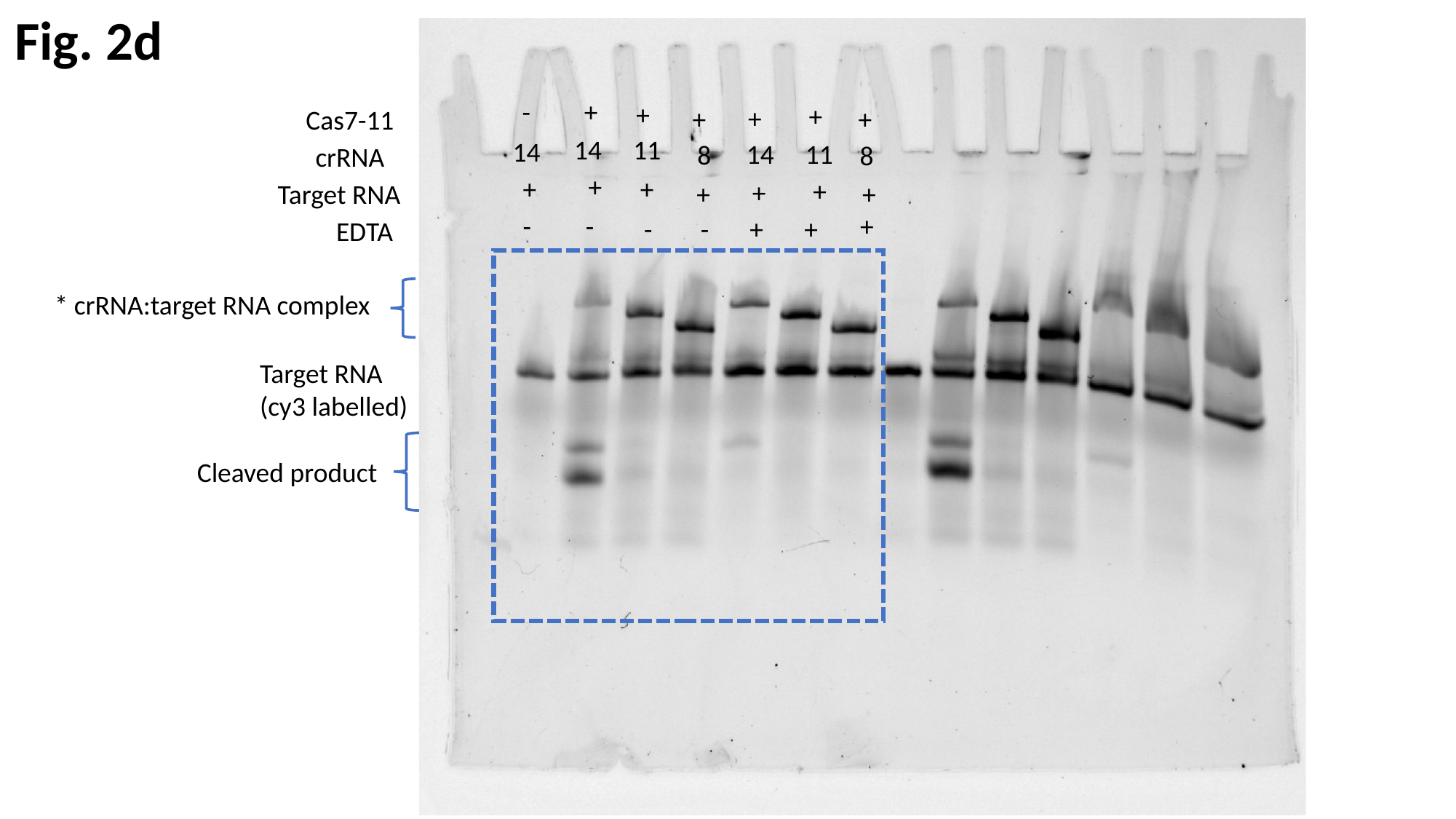

Fig. 2d
-
+
+
+
+
+
+
Cas7-11
14
11
14
14
11
8
8
crRNA
+
+
+
+
+
Target RNA
+
+
-
-
+
-
-
+
+
EDTA
* crRNA:target RNA complex
Target RNA
(cy3 labelled)
Cleaved product
