# Supplementary material for: Molecular mechanism of active Cas7-11 in processing CRISPR RNA and interfering target RNA
Source: eLife. 2022 Oct 3;11:e81678. doi: 10.7554/eLife.81678 (PMC9629832; doi:10.7554/eLife.81678)
Supplement: Figure 4—source data 1. [file elife-81678-fig4-data1.zip › Figure 4 source data 1 /Figure 4 source data 1.pptx]

## Slide 1
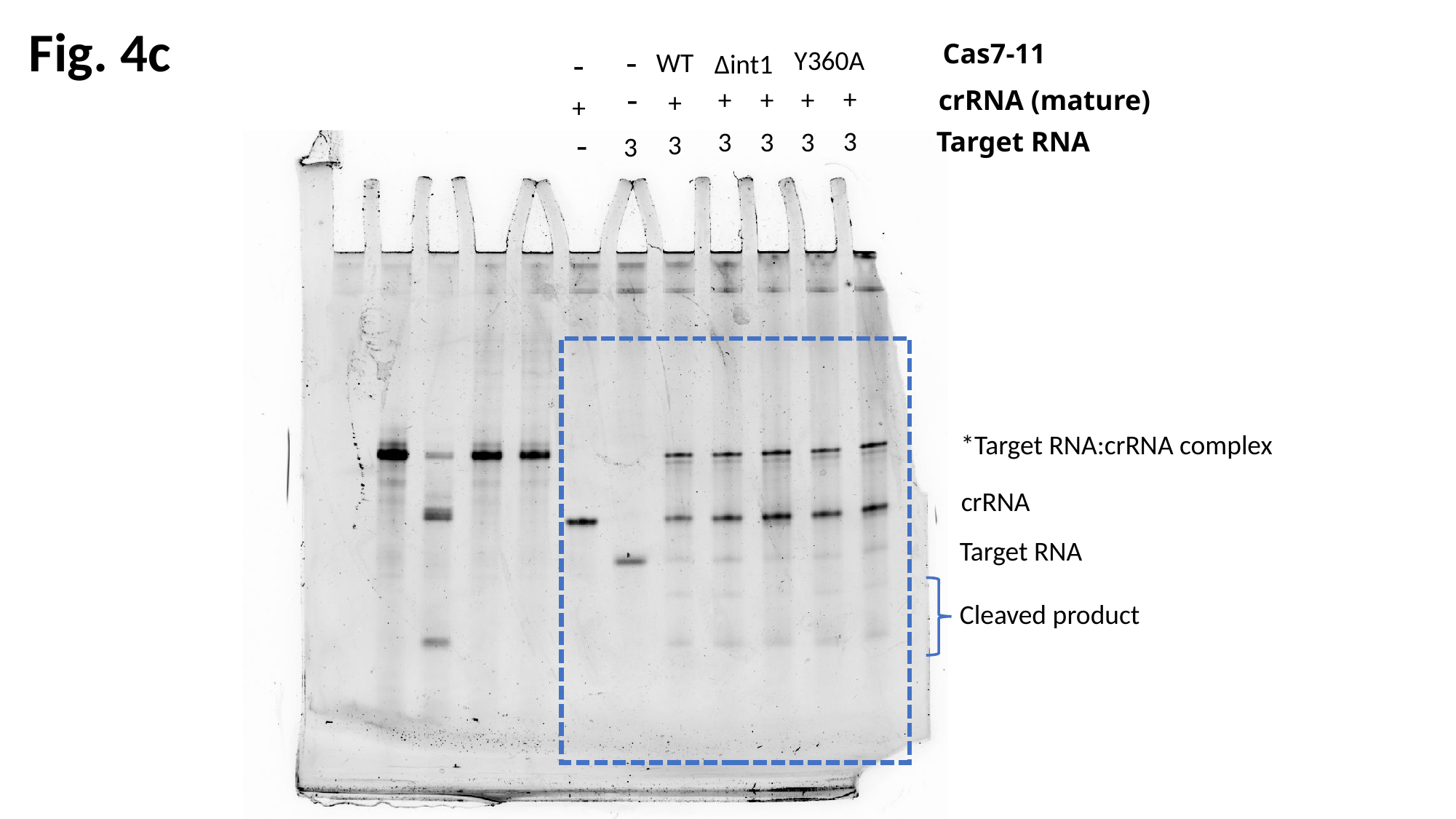

Fig. 4c
Cas7-11
-
-
Y360A
WT
Δint1
-
+
+
+
+
crRNA (mature)
+
+
-
3
3
3
3
Target RNA
3
3
*Target RNA:crRNA complex
crRNA
Target RNA
Cleaved product
