# Supplementary material for: Molecular mechanism of active Cas7-11 in processing CRISPR RNA and interfering target RNA
Source: eLife. 2022 Oct 3;11:e81678. doi: 10.7554/eLife.81678 (PMC9629832; doi:10.7554/eLife.81678)
Supplement: Supplementary file 1. — Related to Figures 2—4. [file elife-81678-supp1.docx]

**Supplementary Table 1.** Data acquisition and processing parameters. Related to Figures 2-4

| Microscope | Titan Krios |
| --- | --- |
| Detector | Gatan K3 |
| V oltage | 300 kV |
| Electron Source | Field Emission Gun |
| Collecting Mode | Counting |
| Dose Rate (e-/Å2) | 60.07 |
| Defocus range (μm) | -1.1 to -2.5 |
| Nominal Magnification | 105000x |
| Frames collected per exposure | 75 |
| Frame alignment Software | MotionCor2 |
| CTF parameter estimation Software | Gctf |
| Total number of raw images collected | 4177 |
| Number of images used for particle picking | 4168 |
| Initial particles picked | 1,301,452 |
| 2D classification software | Cryo-SPARC |
| Final reconstruction software | Relion 4.0 |
| Applied symmetry | C1 |
| Number of particles contributed for the final reconstruction | 226320 |
| Resolution method | FSC 0.143 cut-off |
| Map resolution (Å) | 2.82 |
| Local resolution determining Software | Relion 4.0 |
| Map Visualization software | Pymol/Chimera/ Chimera X/ Coot |
| Deposit EMDB code | 27138 |
| **Refinement parameters** |  |
| CC (map_model) (mask) | 0.83 |
| RMSD (Bond lengths/Bond angles) | 0.006 Å /0.739° |
| Ramachandran plot (%) (Outlier/Allowed/Favored) | (0.0/5.2/94.8) |
| Cβ Outliers (%) | 0.0 |
| MolProbity Score | 2.26 |
| Clash score | 25.8 |
| Rotamer outliers (%) | 0.0 |
| ADP (B-fator) Protein (min/mask/mean)  Nucleotides (min/mask/mean)  Zn (min/mask/mean) | Protein 0.00/74.8/31.5  Nucleotides 0.00/131.1/40.9  Zn 69.9/93.5/80.5 |
| dFSC model (0/0.143/0.5) | 2.5/2.6/2.8 |
| Deposit PDB code | 8D1V |
