# Supplementary material for: Molecular mechanism of active Cas7-11 in processing CRISPR RNA and interfering target RNA
Source: eLife. 2022 Oct 3;11:e81678. doi: 10.7554/eLife.81678 (PMC9629832; doi:10.7554/eLife.81678)
Supplement: Supplementary file 2. — Related to Figures 1—4. [file elife-81678-supp2.docx]

**Supplementary Table 2:** Ribonucleic acid sequences used in this study. Related to Figures 1-4

| Nucleic Acid | Sequence | Source |
| --- | --- | --- |
| DiCas_IVT_1_F  (WT-pre-crRNA) | 5’TAATACGACTCACTATAGGTTGGAAAGCCGGTTTTCTTTGATGTCACGGAACACGTTCTTTGAACCAAGCTTCAAC 3’ | Eurofins |
| DiCas_IVT_1_R  (WT-pre-crRNA) | 5’GTTGAAGCTTGGTTCAAAGAACGTGTTCCGTGACATCAAAGAAAACCGGCTTTCCAACCTATAGTGAGTCGTATTA 3’ | Eurofins |
| pre-crDNA1_F  poly-pre-crRNA 1 | 5’GAAATTAATACGACTCACTATAGGGGAAAAAAAAAAAAAAAAAATTGATGTCACGGAACACGTTCTTTGAACCAAGCTTCAAC 3’ | Eurofins |
| pre-crDNA1_R  poly-pre-crRNA 1 | 5’GTTGAAGCTTGGTTCAAAGAACGTGTTCCGTGACATCAATTTTTTTTTTTTTTTTTTCCCCTATAGTGAGTCGTATTAATTTC 3’ | Eurofins |
| pre-crDNA2_F  poly-pre-crRNA 2 | 5’GAAATTAATACGACTCACTATAGGGGAAAAAAAAAAAAAAATTGATGTCACGGAACACGTTCTTTGAACCAAGCTTCAAC 3’ | Eurofins |
| pre-crDNA2_R  poly-pre-crRNA 2 | 5’GTTGAAGCTTGGTTCAAAGAACGTGTTCCGTGACATCAATTTTTTTTTTTTTTTCCCCTATAGTGAGTCGTATTAATTTC 3’ | Eurofins |
| pre-crDNA3_F  poly-pre-crRNA 3 | 5’GAAATTAATACGACTCACTATAGGGGAAAAAATTGATGTCACGGAACACGTTCTTTGAACCAAGCTTCAAC 3’ | Eurofins |
| pre-crDNA3_R  poly-pre-crRNA 3 | 5’GTTGAAGCTTGGTTCAAAGAACGTGTTCCGTGACATCAATTTTTTCCCCTATAGTGAGTCGTATTAATTTC 3’ | Eurofins |
| Pre-crDNA4_F  (Csb-pre-crRNA) | 5’GAAATTAATACGACTCACTATAGGGTTATGAAACAAGAGAAGGACTTAATGTCACGGTACCGTTCTTTGAACCAAGCTTCAAC 3’ | Eurofins |
| pre-crDNA4_R  (Csb-pre-crRNA) | 5’GTTGAAGCTTGGTTCAAAGAACGGTACCGTGACATTAAGTCCTTCTCTTGTTTCATAACCCTATAGTGAGTCGTATTAATTTC 3’ | Eurofins |
| Target RNA | 5’GGAGUUGAAGCUUGGUUCAAAGAACGUAUCAAGAGCA 3’ | IDT |
| 5’ Cy3 Target RNA | 5’Cy3 GGAGUUGAAGCUUGGUUCAAAGAACGUAUCAAGAGCA 3’ |  |
| Deoxy Target RNA | 5’GGAGUUGAAGCUUGGUUd**C**AAAGAd**A**CGUAU 3’ | IDT |
| PFS less target RNA  (target-RNA2) | 5’GGAGUUGAAGCUUGGUUCAAAGAACGUAU 3’ |  |
| Mature crRNA | 5’UGAUGUCACGGAACACGUUCUUUGAACCAAGCUUCAAC 3’ | IDT |
| -16 deoxy pre crRNA | 5’GGUUGGAAAGCCGGUUUUCTUUGAUGUCACGGAACACGUUCUUUGAACCAAGCUUCAAC 3’ | IDT |
| -15 deoxy pre crRNA | 5’GGUUGGAAAGCCGGUUUUCUTUGAUGUCACGGAACACGUUCUUUGAACCAAGCUUCAAC 3’ | IDT |
| -14 deoxy pre crRNA | 5’GGUUGGAAAGCCGGUUUUCUUTGAUGUCACGGAACACGUUCUUUGAACCAAGCUUCAAC 3’ | IDT |
| 5’ truncated RNA1 | UUCAAAGAACGU | IDT |
| 5’ truncated RNA2 | UUGGUUCAAAGAACGU | IDT |
| 3’ truncated RNA1 | GUUGAAGCUUGGUUCAAAGAACG | IDT |
| 3’ truncated RNA2 | GUUGAAGCUUGGUUCAAA | IDT |
| Cas7 H43A F | TGCCCGTTGGGCCCGCAACAAGAAAGATAAC | Eurofins |
| Cas7 H43A R | AACGCTTGGCCACGCACA | Eurofins |
| Cas7 Y360A F | GTGAGGCGCTTGCTTTGAAGAGTAAG | Eurofins |
| Cas7 Y360A R | CAAGCTTCTCACAAAACTCGC | Eurofins |
| 711 Δ int1 F | CGCGGGCGGTGACCTGAAAGAGAACGAG | Eurofins |
| 711 Δ int1 R | CTGCCACCGCCAGTCTCGGAAAACTTTTGAATG | Eurofins |
| 711 UGA F  (fC7L.1 mutant) | TGAGCGGCCGCACTCGAG | Eurofins |
| 711-D238R  (fC7L.1 mutant) | GTCGAAGCGGATGACGCAC | Eurofins |
